# Metastatic Hepatocellular Carcinoma in a Patient with Crohn's Disease Treated with Azathioprine and Infliximab: A Case Report and Literature Review

**DOI:** 10.1155/2014/340836

**Published:** 2014-12-22

**Authors:** Kyle J. Fortinsky, Ali Alali, Khursheed Jeejeebhoy, Sandra Fischer, Morris Sherman, Scott Fung

**Affiliations:** ^1^Department of Medicine, Toronto General Hospital, 200 Elizabeth Street, Toronto, ON, Canada M5G 2C4; ^2^Department of Medicine, Home Nutrition, St. Michael's Hospital, Toronto, ON, Canada M5B 1W8

## Abstract

Hepatocellular carcinoma most commonly occurs in patients with underlying liver disease or cirrhosis. We describe a case of hepatocellular carcinoma in a 34-year-old man with Crohn's disease treated with azathioprine and infliximab. The patient had no history of liver disease and a complete autoimmune and viral workup was unremarkable. Unfortunately, the patient developed widespread metastatic disease and passed away 5 months after his initial diagnosis. The mechanism of hepatocellular carcinoma in patients' with Crohn's disease is poorly understood and may include both autoimmunity and treatment-related complications. Previous case reports suggest the possibility of a concerning association between azathioprine therapy and the development of hepatocellular carcinoma in patients with Crohn's disease. Clinicians may consider early imaging in patients with Crohn's disease presenting with concerning symptomatology or abnormal liver enzymes, especially in those being treated with azathioprine alone or in combination with infliximab. Future research may help to uncover additional risk factors for this exceedingly rare diagnosis in this patient population.

## 1. Introduction

Hepatocellular carcinoma (HCC) is one of the most commonly diagnosed cancers worldwide [1]. Most patients who develop HCC have cirrhosis, while the minority often have evidence of underlying liver disease [2]. While patients with Crohn's disease (CD) are at increased risk of developing certain cancers (e.g., lymphoma, small bowel, and colon cancer), the incidence of HCC in CD is exceedingly rare [3]. There are currently only ten reported cases of HCC in patients with CD in the English literature [4–13]. Eight of the cases describe patients treated with azathioprine, two of whom were on concurrent infliximab therapy. We are reporting a case of metastatic HCC in a CD patient with no known liver disease who was treated with combination therapy of azathioprine and infliximab. The potential role of autoimmunity, azathioprine, and infliximab in the development of HCC is discussed.

## 2. Case Presentation

A 34-year-old Caucasian man with a 25-year history of CD was admitted to hospital for evaluation of a newly discovered liver mass. His past medical history was significant for an ileocolic resection when he was 14 years old and a proctocolectomy with end ileostomy when he was 22 years old for severe colonic disease resistant to medical therapy. At the age of 28, he developed peristomal pyoderma gangrenosum and seronegative polyarthritis for which therapy with azathioprine 1.5 mg/kg daily and infliximab 5 mg/kg every 6 weeks was initiated. For the previous 6 years, these doses remained the same and controlled his symptoms. He was otherwise well and took no additional medications. Born and raised in Canada, he worked as a structural engineer and was married without any children. He denied any smoking or alcohol or illicit drug use and had no family history of inflammatory bowel disease, liver disease, or malignancy. He had regular visits at his general practitioner and gastroenterologist. His last abdominal ultrasound was performed three years prior to presentation and was entirely normal. More recently, three months prior to presentation, he had routine blood work including liver enzymes as well as a gastroscopy and ileoscopy that were entirely normal. Unfortunately, over the ensuing months he developed progressive epigastric pain, nausea, fatigue, and 20 kg weight loss. Blood work revealed marked elevations in his transaminases and an abdominal ultrasound revealed a large liver mass. The patient was referred to our tertiary care academic institution to confirm the diagnosis and assist with management.

On presentation to hospital, the patient appeared well without any evidence of jaundice or stigmata of chronic liver disease. His liver enzymes and alpha-fetoprotein level were markedly elevated, while his liver function was normal (Table 1).

**Figure 1:**
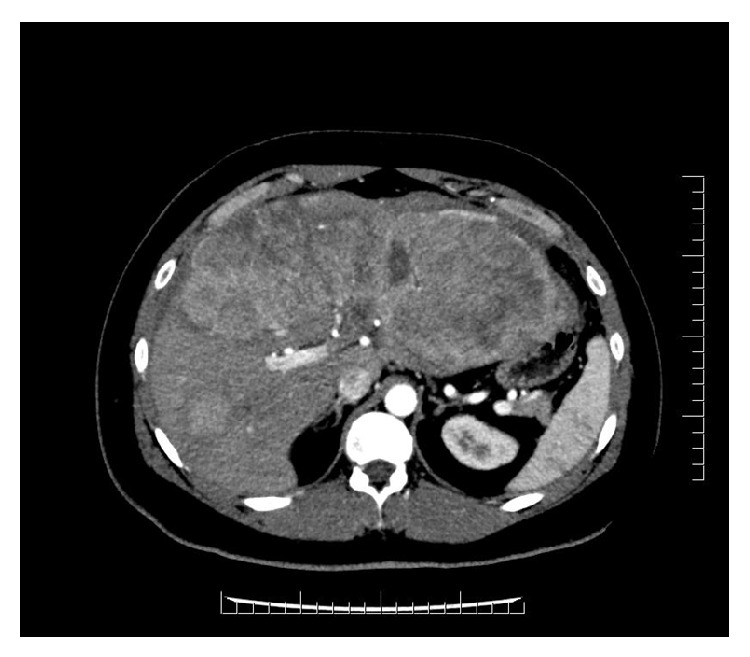
Hepatocellular tumor measuring 24 cm with portal vein tumor thrombosis and satellite tumors in the right lobe.

**Figure 2:**
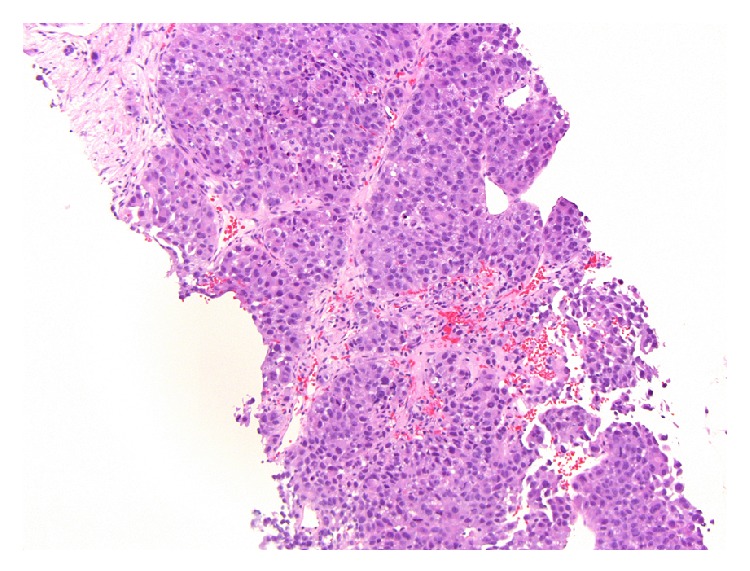
A biopsy from a liver mass reveals a malignant neoplasm formed by large atypical polygonal cells with trabecular pattern of growth (hematoxylin and eosin, ×100).

**Figure 3:**
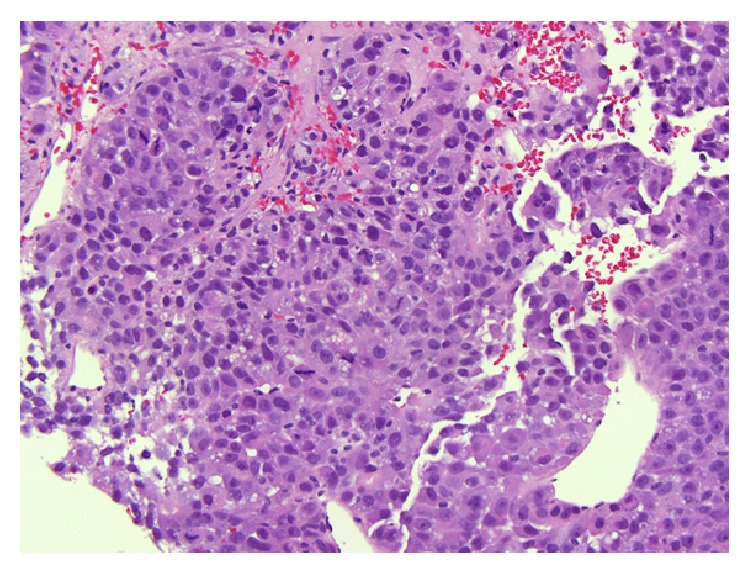
Tumor cells have abundant cytoplasm, high nucleus-to-cytoplasm ratio, irregular nuclear contours, and prominent nucleoli (hematoxylin and eosin, ×200).

An abdominal CT scan revealed a 24-centimeter mass in his left hepatic lobe with tumor thrombosis involving the left portal vein and nodular masses in the right lobe (Figure 1). A CT of the chest and pelvis did not reveal evidence of distant metastases. A complete liver disease workup was performed and included hepatitis B and C serology, alpha-1 antitrypsin, anti-nuclear antibody, smooth muscle antibody, anti-neutrophil cytoplasmic antibody, anti-myeloperoxidase antibody, proteinase 3 antibody, complement levels, immunoglobulin levels, iron studies, and ceruloplasmin. All laboratory results were entirely unremarkable. Multiple liver biopsies were performed which confirmed the diagnosis of HCC but were unable to identify any underlying liver tissue (Figures 2 and 3).

The case was presented at multidisciplinary rounds that included representatives from hepatology, medical oncology, radiation oncology, and surgical oncology. The diagnosis, prognosis, and treatment options were discussed with the patient and his family. Given the size of the tumor, neither surgery nor liver transplantation was a viable option, and the patient was commenced on Sorafenib therapy and was provided with transcatheter arterial chemoembolization (TACE). The decision was made to discontinue his azathioprine and infliximab since they may have been contributing to the rapid tumor growth. Immediately after TACE, the patient remained well without any evidence of liver dysfunction or distant metastases. He was discharged home with close follow-up from medical oncology and hepatology. Unfortunately, over the ensuing three months, he developed progressive liver dysfunction and sustained a massive oropharynx bleed that required multiple blood transfusions. It was determined that the severe bleeding episode was in the context of a tumor involving his right mandible that eroded into his oropharynx. Repeat imaging revealed widespread metastatic disease involving his lungs, adrenals, thoracic spine, maxilla, and mandible. Unfortunately, while in hospital the patient developed progressive liver and renal failure and was in considerable pain from his widespread metastatic disease. The patient was provided with palliative care and passed away approximately five months after his initial diagnosis with HCC. The family declined an autopsy.

## 3. Discussion

This case represents only the eleventh report of hepatocellular carcinoma in Crohn's disease [4–13]. Furthermore, this case represents only the third case of HCC in patients with CD on combination therapy with azathioprine and infliximab. Intriguingly, eight of the ten CD patients who developed HCC were exposed to azathioprine therapy [7]. The remaining two CD patients with HCC were never exposed to azathioprine therapy but were both ultimately found to have evidence of underlying liver disease [10, 12]. There are no reported cases of HCC in CD patients who have no underlying liver disease and who were not exposed to azathioprine therapy. It remains unclear whether there is a direct association between azathioprine and HCC, although it appears the CD itself may be playing a role as well. Indeed, there are no cases of HCC in patients without CD who are being treated with azathioprine for another indication. Given the scarcity of cases, the potential additive effect of infliximab in combination with azathioprine is largely unknown with regard to malignancy risk.

Azathioprine, a thiopurine drug, is commonly used for its anti-inflammatory properties in the management of patients with CD. Although azathioprine works well for CD either alone or in combination with a tumor necrosis factor inhibitor, thiopurine drugs may induce cell mutation and affect DNA repair mechanisms, which can put individuals at increased risk of malignancy [14, 15]. Azathioprine therapy for inflammatory bowel disease has been linked to various cancers including lymphoma, urinary tract malignancies, and skin cancers [16–18]. While large cohort studies have failed to show any association of azathioprine and hepatocellular carcinoma, there is biological plausibility to this relationship given azathioprine's known hepatotoxicity and mutagenicity. It is possible that these studies are underpowered to detect an association because of the extremely low incidence of HCC in these populations combined with the potential inaccuracies of using national drug database information [16, 17]. The current case and the previous ones published in the literature do raise the suspicion of a rare association between azathioprine and HCC in CD patients.

Many liver diseases have been associated with CD including nonalcoholic liver disease [19], primary sclerosing cholangitis [20], aseptic liver abscesses [21], abnormal liver function [22], and inflammatory pseudotumors [23]. One hypothesis is that the gut-liver axis may be playing a role in the evolution of liver diseases in the setting of CD [24]. More specifically, hepatic injury in CD may be mediated by the inappropriate recruitment of mucosal T cells to the liver as a result of aberrantly expressed endothelial-cell adhesion molecules and chemokines that are routinely only found in the gut [25]. Recently, it was discovered that patients with autoimmune diseases are at increased risk of several hepatobiliary cancers [26]. There are presently no reports of HCC in patients receiving azathioprine therapy for a disease other than CD, thus suggesting that CD itself may be a factor in the development of HCC irrespective of azathioprine.

The additive role of infliximab in the development of HCC in our patient is unclear. To date, there are no case reports of HCC in patients with or without CD who were treated with infliximab alone. Two large-scale epidemiological studies found no relationship between infliximab use and malignancy [27, 28]. As such, in both our patient and the two previous cases of HCC in CD patients on combination therapy, it is possible that infliximab is an innocent bystander.

## 4. Conclusion

Large-scale studies have failed to show an association between azathioprine therapy and HCC in CD, although, given the rarity of this disease, it may be extremely difficult to demonstrate. Nevertheless, it remains concerning that all reported cases of HCC in CD without underlying liver disease seem to be associated with azathioprine therapy. One hypothesis may be that CD generates an environment of inflammation in the gut-liver axis that could prime the liver for dysplasia. Azathioprine, in this inflammatory environment, could cause injury to the hepatocytes eventually creating cancerous cells that may propagate in the setting of a compromised immune system.

The current case illustrates hepatocellular carcinoma as a rare and potentially fatal diagnosis in a patient with Crohn's disease. The etiology of HCC in patients with CD is poorly understood and requires further investigation. Clinicians should consider early imaging in patients with CD presenting with concerning symptomatology or abnormal liver enzymes, especially in those being treated with azathioprine alone or in combination with infliximab.

## Figures and Tables

**Table 1:** Pertinent laboratory values on admission.

Laboratory parameter	Value on admission	Reference range
Hemoglobin (g/L)	132	120–165
White-cell count (×10^9^/L)	7.1	4–11
Platelet count (×10^9^/L)	328	150–450
Creatinine (umol/L)	88	40–100
Alanine aminotransferase (IU/L)	82	3–36
Aspartate aminotransferase (IU/L)	163	0–35
Alkaline phosphatase (IU/L)	279	35–100
Alpha fetoprotein (ug/L)	3307	0–20
Total bilirubin (umol/L)	16	0–26
International normalized ratio	1.1	0.9–1.2
